# Attack behaviour in naive gyrfalcons is modelled by the same guidance law as in peregrine falcons, but at a lower guidance gain

**DOI:** 10.1242/jeb.238493

**Published:** 2021-03-02

**Authors:** Caroline H. Brighton, Katherine E. Chapman, Nicholas C. Fox, Graham K. Taylor

**Affiliations:** 1 Department of Zoology, University of Oxford, Oxford, OX1 3SZ, UK; 2 Wingbeat Ltd, Carmarthen, SA33 5YL, UK

**Keywords:** Aerial pursuit, Optimal guidance, Proportional navigation, Proportional pursuit, *Falco rusticolus*, *Falco peregrinus*

## Abstract

The aerial hunting behaviours of birds are strongly influenced by flight morphology and ecology, but little is known of how this relates to the behavioural algorithms guiding flight. Here, we used GPS loggers to record the attack trajectories of captive-bred gyrfalcons (*Falco rusticolus*) during their maiden flights against robotic aerial targets, which we compared with existing flight data from peregrine falcons (*Falco peregrinus*). The attack trajectories of both species were well modelled by a proportional navigation (PN) guidance law, which commands turning in proportion to the angular rate of the line-of-sight to target, at a guidance gain *N*. However, naive gyrfalcons operate at significantly lower values of *N* than peregrine falcons, producing slower turning and a longer path to intercept. Gyrfalcons are less manoeuvrable than peregrine falcons, but physical constraint is insufficient to explain the lower values of *N* we found, which may reflect either the inexperience of the individual birds or ecological adaptation at the species level. For example, low values of *N* promote the tail-chasing behaviour that is typical of wild gyrfalcons and which apparently serves to tire their prey in a prolonged high-speed pursuit. Likewise, during close pursuit of typical fast evasive prey, PN will be less prone to being thrown off by erratic target manoeuvres at low guidance gain. The fact that low-gain PN successfully models the maiden attack flights of gyrfalcons suggests that this behavioural algorithm is embedded in a guidance pathway ancestral to the clade containing gyrfalcons and peregrine falcons, though perhaps with much deeper evolutionary origins.

## Abstract (precis)

**Highlighted Article:** Naive gyrfalcons attacking aerial targets are modelled by the same proportional navigation guidance law as peregrine falcons, but with a lower navigation constant that promotes tail-chasing rather than efficient interception.

## INTRODUCTION

Raptorial feeding is a complex mode of foraging behaviour, the success of which hinges on intercepting a target whose own success hinges on evading capture. Aerial pursuit in particular is one of the most challenging behaviours that organisms perform, but also one of the simplest to characterize. A dyadic interaction, for example, is minimally described by the trajectories of a pair of interacting particles representing the predator and its prey. This level of description lends itself to an algorithmic approach, in which a mathematical rule – in this case, a particular kind of behavioural algorithm known as a guidance law – is used to connect sensory input to motor output (Hein et al., 2020). As there are only a limited number of ways in which one particle can be steered to intercept another, this algorithmic approach lends itself in turn to a rigorous comparative analysis of behaviour across different taxa, locomotor modes and spatiotemporal scales. Key research questions include: what sensory information is used to guide interception, and how?; for what function is the attacker's guidance algorithm optimized?; and how is this behavioural algorithm acquired? Here, we addressed these questions for a sample of naive gyrfalcons (*Falco rusticolus* Linnaeus 1758), which are the largest of all falcons, and hence one of the largest extant predators specializing in aerial interception.

Gyrfalcons are closely related to peregrine falcons (*Falco peregrinus* Tunstall 1771), whose attack trajectories are well modelled (Brighton et al., 2017) by a guidance law called proportional navigation (PN) (Fig. S1). A pure PN guidance law commands turning at an angular rate 
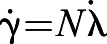
, where 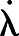 is the angular rate of the attacker's line-of-sight to target, and where the guidance gain *N* is called the navigation constant and is assumed to be fixed within an attack. In contrast, the attack trajectories of Harris’ hawks (*Parabuteo unicinctus*) are best modelled by a mixed PN+PP guidance law, 
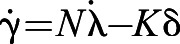
, which combines a PN element with a proportional pursuit (PP) element, −*K*δ, commanding turning in proportion to the deviation angle δ between the attacker's velocity vector and its line-of-sight to target (Brighton and Taylor, 2019). In this case, a PN+PP guidance law with guidance constants *N*=0.7 and *K*=1.2 s^−1^ modelled the observed flights more closely than a PN or PP guidance law in which *N* or *K* was allowed to vary between flights (Brighton and Taylor, 2019). The PN guidance law of peregrine falcons promotes short-cutting towards the eventual point of intercept, making it well suited to intercepting non-manoeuvring targets in open environments (Brighton et al., 2017). In contrast, the PN+PP guidance law of Harris' hawks promotes tail-chasing directly after the target, making it better suited to close pursuit through potentially cluttered environments (Brighton and Taylor, 2019). Hawks (Accipitridae) and falcons (Falconidae) are thought to have diverged >60 Mya (Jarvis et al., 2014; Prum et al., 2015), so on phylogenetic grounds we might reasonably expect the attack behaviour of gyrfalcons to be better modelled by the PN guidance law of peregrine falcons than by the PN+PP guidance law of Harris' hawks.

Peregrine falcons and gyrfalcons are both adapted to open environments, hunting mainly avian prey, which may be knocked down in flight (Garber et al., 1993), struck on the ground before taking flight (Bengtson, 1971) or forced to the ground after a long chase (Cade, 1982; Woodin, 1967). However, whereas peregrine falcons will often dive from altitude in a high-speed stoop (Cresswell, 1996), gyrfalcons rarely stoop in the wild and almost always hunt close to the ground (Cade, 1982; Garber et al., 1993). Gyrfalcons are most often recorded performing low surprise attacks initiated from a perch or from ridge soaring (Cade, 1982; Garber et al., 1993; Potapov and Sale, 2005; White and Nelson, 1991; White and Weeden, 1966), but if not immediately successful then they will commonly enter the prolonged tail-chase that is typical of this species (Cade, 1982; Pennycuick et al., 1994). Similar hunting behaviours are also observed in peregrine falcons (Cresswell, 1996), but the distinct speed advantage that peregrine falcons acquire when stooping (Mills et al., 2018, 2019) appears to be lacking in wild gyrfalcons. Such variation in hunting behaviour may be expected to be associated with variation in the underlying guidance law.

Peregrine falcon attacks are best modelled by a range of values of *N* (median: 2.6; 1st, 3rd quartiles: 1.5, 3.2) lower than the interval 3≤*N*≤5 that is typical of missile applications, but close to the optimum minimizing total steering effort in the classical linear-quadratic formulation of the optimal guidance problem (Brighton et al., 2017). This theory predicts that *N*=3*v*_c_/(*v*cosδ) is optimal for attacks on non-manoeuvring targets, where *v*_c_ is the speed at which the attacker closes range on its target, and *v* is the attacker's groundspeed (Shneydor, 1998; Siouris, 2004). In words, the optimal value of *N* is proportional to the ratio *v*_c_/(*v*cosδ), which expresses how effectively the attacker closes range on its target (*v*_c_, representing the difference between the speed of the attacker's approach and the speed of the target's retreat) in relation to its own motion towards the target (*v*cosδ, representing the speed of the attacker's approach). Hence, whereas *N*=3 is optimal for attacks on stationary targets (where *v*_c_=*v*cosδ), *N*<3 is optimal for attacks on retreating targets (where *v*_c_<*v*cosδ), at a value which depends on the relative speed of the target and attacker. In a high-speed stoop, for instance, the target's speed may be negligible compared with that of its attacker, such that *v*_c_/(*v*cosδ)≈1 making *N*≈3 optimal (Mills et al., 2018; Mills et al., 2019). Conversely, in a prolonged tail chase in which the target flees at a similar speed to the attacker, much lower values of *N*<3 may be optimal. We therefore hypothesize that gyrfalcons will use PN guidance to intercept targets but will do so using a lower value of *N* than peregrine falcons.

## MATERIALS AND METHODS

To test these hypotheses, we used a combination of empirical measurements and computational modelling to identify the guidance law used by naive captive-bred gyrfalcons chasing robotic aerial targets. Our observations were recorded on the first attack flights that the birds had ever made against aerial targets, and therefore reflect as closely as possible the innate form of the underlying sensorimotor pathway with no prior opportunity for adapting this through experience.

### Animals

We observed 23 naive captive-bred gyrfalcons, comprising 19 pure gyrfalcons (*F. rusticolus*) and 4 gyrfalcon–saker falcon hybrids (7/8th *Falco* *rusticolus*×1/8th *Falco* *cherrug*), chasing robotic aerial targets during their first flight sessions. This sample contained only naive first-year birds that had not previously flown after aerial targets, except for during a single flight against a swung lure immediately beforehand. This work received approval from the Animal Welfare and Ethical Review Board of the Department of Zoology, University of Oxford, in accordance with University policy on the use of protected animals for scientific research, permit no. APA/1/5/ZOO/NASPA, and is considered not to pose any significant risk of causing pain, suffering, damage or lasting harm to the animals.

### Experimental protocol

**Fig. 1.**
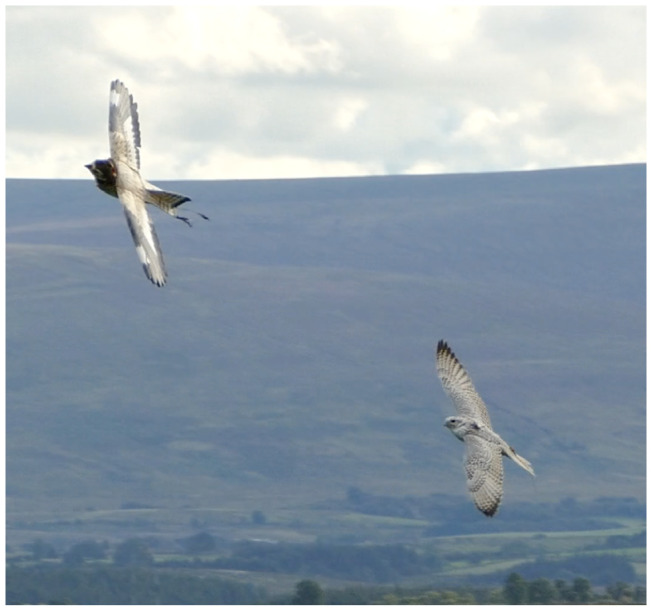
**Cropped frame from a video of a typical chase involving a gyrfalcon and a ‘Rokarrowan’ Roprey model.** Note the proximity of the attacker to its target, and their similar bank angles, which are characteristic of the tail-chasing behaviour that we observed.

**Fig. 2.**
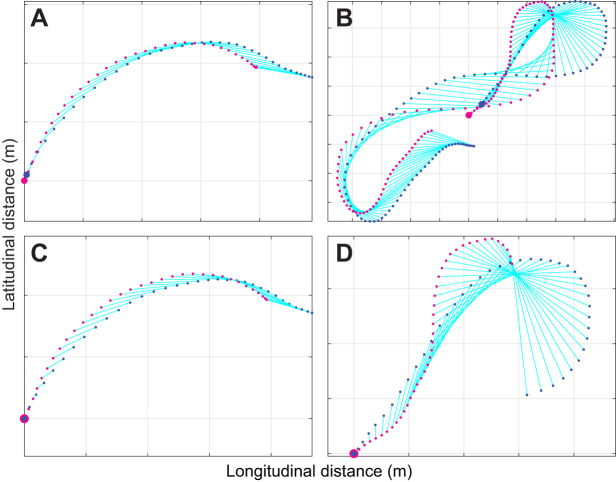
**Two-dimensional (2D) GPS trajectories for example short dashes and extended chases.** (A,B) Example trajectories showing the entirety of a (A) short dash and (B) an extended chase, showing the lines of sight (cyan lines) between the gyrfalcon (blue points) and Roprey (magenta points) at each sample point. Note the small discrepancy in the estimated position of target and attacker at the point of intercept (enlarged sample points), expected because of the positioning error associated with GPS receivers (see Materials and Methods). (C,D) Trajectories for the terminal phase of the same two flights (C, short dash; D extended chase; see Fig. 4G,M for modelling), after shifting the attacker's trajectory to correct for this positioning error. Gridlines are at 10 m spacing.

Flight trials were carried out on open moorland at Watch Hill, Wealside Farm, Northumberland, UK, in winds gusting from 4 to 7 m s^−1^. The birds were recorded chasing a remotely piloted, ducted fan ‘Roprey’ model with a food reward strapped to its dorsal surface (Wingbeat Ltd, Carmarthen, UK; Fig. 1). Each bird carried a BT-Q1300 GPS receiver (QStarz International, Taipei, Taiwan) logging position and groundspeed at 5 Hz. The GPS receiver was carried dorsally on a Trackpack harness (Marshall Radio Telemetry, Salt Lake City, UT, USA), giving a total load of 0.015 kg. An identical GPS logger was fixed inside the body compartment of the Roprey, and the flights were filmed using a handheld Lumix DMC-FZ1000 camera recording 4k video at 25 frames s^−1^ (Panasonic Corporation, Osaka, Japan). Each flight trial began as the falconer unhooded the bird on their fist, with the Roprey held ∼20 m upwind. The Roprey was launched as soon as the bird took off, or sometimes just beforehand; if not caught immediately (e.g. Fig. 2A), it was flown through a series of evasive turns (e.g. Fig. 2B). The trial ended when the bird first intercepted the Roprey. If the bird knocked the Roprey with its talons, then the pilot brought it safely to the ground; if the bird bound to the Roprey, then the motor was cut, and an airbrake was deployed to prevent the pair from drifting. In a few cases, the Roprey crash-landed before finally being intercepted by the bird. After disregarding 3 flights in which the bird did not intercept the target, and another 7 flights in which the GPS logger was lost during the session, we were left with an initial sample of 28 flights from 19 naive gyrfalcons.

### Data synchronization and error analysis

We used the GPS time signal to synchronize the two GPS data streams to within ±0.1 s, having linearly interpolated a small number of dropped datapoints. We matched the synchronized GPS data to the video with reference to take-off and landing, and used the video to identify the time of first intercept. We then shifted the entire GPS trajectory of the bird so as to match its estimated position at first intercept to that of the target (Fig. 2). This adjustment was necessary to remove positional bias due to GPS receiver clock error, which is such that at the nominal horizontal positioning accuracy of our GPS receivers (<3.0 m circular error probable), we would expect 50% of position estimates of a co-located pair of receivers to be separated by ≥4.8 m under an isotropic gaussian error model. Receiver clock error varies slowly once the receiver clock time estimate has settled after start-up, so has no significant effect on the measurement of changes in position over short time scales: in fact, we have shown empirically that our receivers have a precision of the order of 0.1 m for changes in position occurring over intervals of the order of 10 s (Brighton et al., 2017). Even so, there were 8 flights for which the discrepancy in the horizontal position estimates of the bird and target at intercept exceeded the 95th percentile expected at the nominal accuracy of the receivers, which indicates a higher than expected positioning error in one or both receivers. Of these 8 flagged flights, 5 were the first flight that we recorded after receiver start-up at the beginning of a logging session, which suggests that the receiver clock estimate had not been given sufficient time to settle at the start of all 7 logging sessions (Fisher's exact test: *P*=0.01). We therefore dropped these 8 flagged flights from the analysis, leaving a final sample of 20 flights by *n*=13 gyrfalcons (Table S1). Among these 20 remaining flights, the discrepancies in the position estimates of bird and target at intercept (median: 5.5 m; interquartile range, IQR: 8.0–3.1 m) were distributed as expected at the nominal accuracy of the receivers (median: 4.8 m; IQR: 7.3–2.8 m), albeit with no extreme outliers.

### Trajectory modelling

We simulated the birds' measured flight trajectories in Matlab, by predicting their turn rate 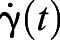 in response to the target's measured trajectory using a guidance law of the form:(1)
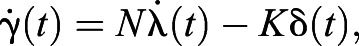
where 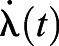 is the angular rate of the line-of-sight to target, where δ(*t*) is the deviation angle between the target and the attacker's velocity vector, and where *N* and *K* are constants. Angles and angular rates are defined in vector form in the three-dimensional (3D) case, but can be treated as scalars in the two-dimensional (2D) case. Eqn 1 necessarily ignores the effects of any sensorimotor delay, on the basis that this is expected to be comparable to the ±0.1 s uncertainty in the synchronization of the GPS data streams (see Brighton and Taylor, 2019). In the special case that *K*=0, Eqn 1 reduces to a pure PN guidance law, whereas in the special case that *N*=0, Eqn 1 reduces to a pure PP guidance law. We refer to the general case in which *K*≠0 and *N*≠0 as mixed PN+PP guidance. We used a forward Euler method to simulate each flight under either PN, PP or PN+PP guidance, initializing the simulation using the bird's measured position and velocity at some given start point, and matching the bird's simulated flight speed to its known groundspeed. The method is described further in our work with peregrine falcons and Harris' hawks (Shneydor, 1998; Siouris, 2004), the only difference being the 1/3000 s step size that was used here to ensure that all parameter estimates were accurate at the reported precision (for simulation code, see Supporting Data S1).

Treating each flight independently, we used a Nelder–Mead simplex algorithm to find the value of *N* and/or *K* that minimized the mean prediction error for each flight, defined as the mean absolute distance between the measured and simulated trajectories. Because the bird did not always start chasing the target from the moment it was launched, it was necessary to select the start-point of the simulation by reference to the data. Following the same approach used to model aerial chases in peregrine falcons (Brighton et al., 2017), we ran simulations beginning from all possible start times ≥2.0 s before intercept, reporting the longest 2D simulation (up to a maximum of 20 s) for which the mean prediction error was ≤1.0% of the flight distance modelled. For the 3D case, we used an equivalent error tolerance of 1.2%, to preserve the same tolerance in each dimension. Simulations that failed to model ≥2.0 s of flight time at the specified error tolerance are recorded as unsuccessful, and their parameter estimates are not reported. Hence, because all of the successful simulations were fitted to within the same specified error tolerance, the appropriate figure of merit for each successful simulation is the overall distance or duration of the flight that was modelled at this error tolerance. The process of selecting the start-point of each simulation by reference to the model's performance on the data is objective, but risks capitalizing on chance. To ensure that our inferences were robust to the associated risk of overfitting, we therefore limited ourselves to making contrastive inferences between alternative guidance models and distributional inferences on the population properties of the estimated parameters.

As a further control against the risk of overfitting, we also ran a global analysis in which we identified the unique value of *N* and/or *K* that maximized the total distance of flight fitted in 2D at a 1.0% error tolerance over all of the flights. We did this by using an exhaustive search procedure across all possible start times ≥2.0 s before intercept for each flight (up to a maximum of 20 s), and for all possible values of *N* and *K* on the closed interval from 0 to 2.0 at 0.05 spacing. These globally fitted simulations model less of the data than the independently fitted simulations, so the two analyses are complementary rather than redundant. Specifically, whereas the independently fitted simulations model as much of the data as possible and account for the possibility that the guidance parameters might vary between individuals and flights, the globally fitted simulations eliminate any risk of overfitting at the expense of modelling less of the data on the assumption that the guidance parameters are fixed across individuals and flights.

To facilitate direct comparison with our published results from experienced peregrine falcons (Brighton et al., 2017), we reanalysed the peregrine falcon dataset using exactly the same methods as described above. After excluding 33 flights representing attacks on stationary ground targets, and after rejecting another 9 flights that were flagged as having lower than expected accuracy at the point of intercept by the method above, this yielded a refined sub-sample of 13 flights against aerial targets made by 4 experienced peregrine falcons (*F. peregrinus*). The mixed PN+PP guidance law was not considered in the original analysis (Brighton et al., 2017), so was fitted for the first time here. The only other difference from the original analysis was the refinement of the integration step size used here. This had only a small effect on the simulated trajectories, but sometimes caused a different start point to be selected for the simulations, because of the thresholding associated with finding the longest simulation fitted at the specified error tolerance.

## RESULTS

### The attack trajectories of naive gyrfalcons are well modelled under PN

**Fig. 3.**
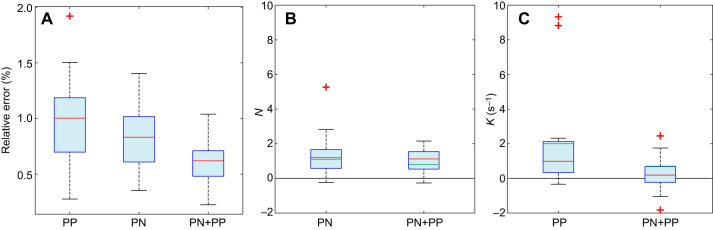
**Box-and-whisker plots comparing model fits and parameter estimates for 2D simulations of 20 flights from *n*=13 gyrfalcons under proportional pursuit (PP), proportional navigation (PN) and mixed (PN+PP) guidance.** (A) Relative error of simulation, showing either the relative error for the longest simulation lasting ≥2 s that met the 1.0% error tolerance threshold for each flight or, if no simulation met this threshold, the minimum relative error achieved on any simulation lasting ≥2 s. Note that whilst the PN+PP simulations fit the flights more closely than either PN or PP, they are almost certainly overfitted (see Results). (B,C) Parameter estimates for the guidance constants *N* and *K* for all successfully modelled flights. Note the variable sign of the parameter estimates for *K* under PN+PP, which confirms that these simulations are overfitted (one outlier for PN+PP not shown). The red line in each box denotes the median value for all flights; the lower and upper bounds of the box denote the 1st and 3rd quartiles; crosses indicate outliers falling >1.5 times the interquartile range beyond the 1st or 3rd quartile; whiskers extend to the farthest datapoints not treated as outliers. The green lines denote the global optimal values of the guidance parameters.

**Fig. 4.**
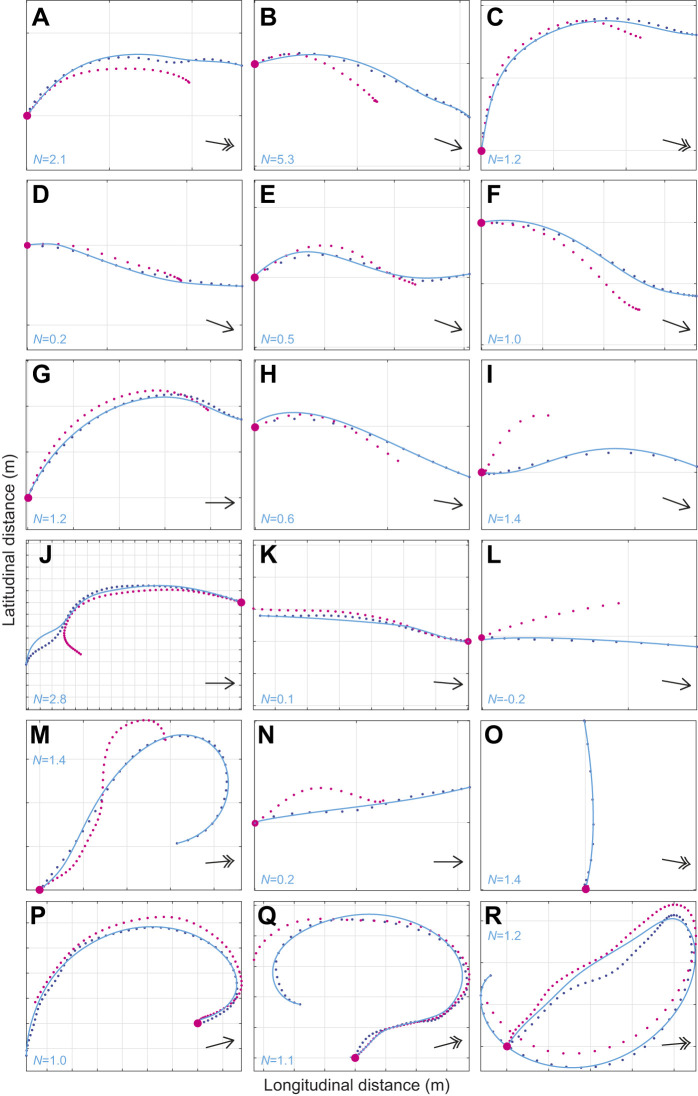
**2D attack trajectories for the 18/20 flights from *n*=13 gyrfalcons that were successfully modelled under PN guidance.** Panels show the measured trajectories of the target (magenta points) and attacker (blue points), overlain with the longest simulation fitted to within 1.0% error tolerance (blue lines) in 2D. The corresponding parameter estimate for *N* is displayed on each plot. Note that among the 9 short dashes (A–I), 7 flights (A–G) are modelled in their entirety from target launch to intercept; the other 9 flights (J–R) each correspond to the terminal phase of an extended chase. Simulations with values of *N* falling beneath the 1st quartile (*N*<0.5) coincide with nearly straight sections of flight (D,K,L,N), for which parameter estimation is unreliable. Simulations with values of *N* falling between the 1st and 3rd quartiles (0.5≤*N*≤1.4) involve a substantial amount of turning that the model successfully explains (C,E–I,M,O–R). Black arrows display mean wind direction; double-headed arrows correspond to wind speeds >20 km h^−1^; gridlines are at 10 m spacing. See Fig. S2 for the remaining 2/20 flights that were not successfully modelled under PN.

The independently fitted simulations under PN successfully modelled 18/20 flights by the naive gyrfalcons, fitting 1127 m and 111.0 s of flight at 1.0% error tolerance in 2D (Fig. 3; Fig. S2, Table S2), with a median guidance parameter estimate of *N*=1.2 (1st, 3rd quartiles: 0.5, 1.4). In contrast, the independently fitted simulations under PP modelled only 14/20 flights successfully, fitting 824 m and 77.4 s of flight at 1.0% error tolerance in 2D (Fig. 3A,C; Table S2), with a median guidance parameter estimate of *K*=0.9 s^−1^ (1st, 3rd quartiles: 0.2, 1.7 s^−1^). It follows that PN is the much better supported of the two pure guidance laws on the basis of the simulations fitted independently to each flight. Moreover, all of the PN simulations with values of *N* falling between the 1st and 3rd quartiles were from flights involving a substantial amount of horizontal turning (Fig. 4C,E–I,M,O–R), which proportional feedback of the line-of-sight rate 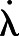 successfully explains. Qualitatively the same pattern was seen in the globally fitted simulations, which successfully modelled 12/20 flights under PN, but only 5/20 flights under PP. Whereas these simulations fitted 628 m and 62.8 s of flight at 1.0% error tolerance at the global optimum of *N*=1.1 for PN, they fitted only 419 m and 36.4 s of flight at the global optimum of *K*=2.0 for PP, so PN is also the much better supported of the two pure guidance laws on the basis of the simulations fitted globally to all flights. Furthermore, the median guidance parameter estimate for the independently fitted PN simulations (*N*=1.2) coincides closely with the global optimum (*N*=1.1), so there is a high degree of consistency in the conclusions drawn from these two complementary approaches to the model fitting.

The independently fitted simulations under mixed PN+PP guidance successfully modelled the terminal phase of all 20 flights, fitting 1358 m and 139.4 s of flight at 1.0% error tolerance in 2D (Fig. 3A; Table S2). However, whilst the addition of a PP term therefore increased the duration of flight fitted by a factor of 1.3 relative to PN, this apparent improvement is almost certainly attributable to overfitting. Specifically, the parameter estimates for *K* were inconsistently signed in the PN+PP simulations (median *K*: 0.2 s^−1^; 1st, 3rd, quartiles: −0.2, 0.7 s^−1^; two-tailed sign test: *P*=0.26; *n*=20 flights; Fig. 3C), despite being positive in most of the successful PP simulations (two-tailed sign test: *P*=0.01; *n*=14 flights; Fig. 3C). This volatility in the sign of the parameter estimates for *K* under mixed PN+PP guidance indicates that any turning behaviour that is not already modelled by the PN element of the PN+PP simulations is not consistently modelled by their PP element either. Hence, given that *K*>0 steers flight towards the target, whereas *K*<0 steers flight away from it, there is no evidence to indicate that a PP element supplements PN in commanding steering towards the target. Conversely, the observation that the parameter estimates for *N* were usually positive in the successful PN simulations (two-tailed sign test: *P*<0.001; *n*=18 flights; Fig. 3B), and likewise in the PN+PP simulations (median *N*=1.1; 1st, 3rd quartiles: 0.5, 1.5; two-tailed sign test: *P*=0.003; *n*=20 flights; Fig. 3B), provides strong positive evidence that steering towards the target is indeed based on feeding back the line-of-sight rate 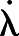 rather than the deviation angle δ. This conclusion is reinforced by the globally fitted simulations maximizing the total distance of flight fitted at 1.0% error tolerance over all flights. Although PN+PP at the global optimum of *N*=0.8 and *K*=0.7 of course fitted more flight than PN at the global optimum of *N*=1.1, the improvement resulting from the addition of another fitted guidance parameter was equivocal. Specifically, whereas the globally fitted PN+PP simulations fitted 759 m and 68.2 s of flight at 1.0% error tolerance under PN+PP, they did so for only 11/20 flights. In contrast, the globally fitted PN simulations fitted 628 m and 62.8 s of flight, and did so for 12/20 flights. Hence, the hypothesis that naive gyrfalcons use PN guidance is well supported relative to the alternatives.

**Fig. 5.**
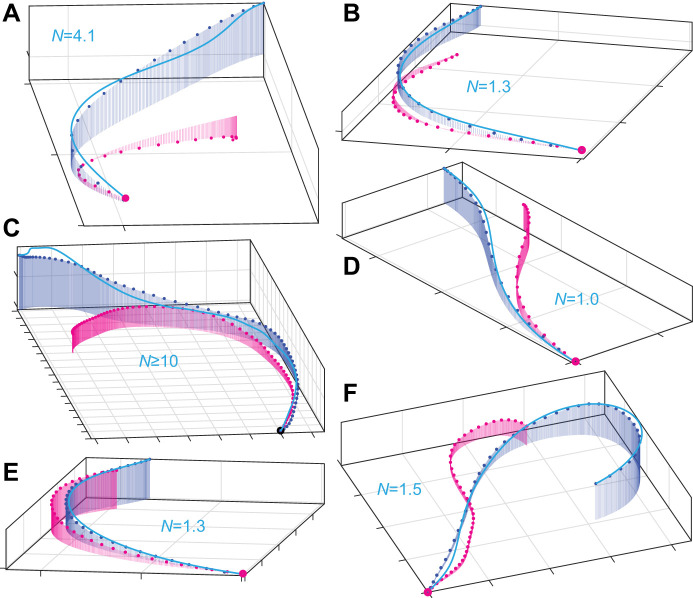
**Three-dimensional (3D) attack trajectories for the subset of 6 gyrfalcon flights involving the greatest altitudinal change among the 12/20 flights that were successfully modelled under PN guidance.** (A–F) The measured trajectories of the target (magenta points) and attacker (blue points), overlain with the longest simulation fitted to within 1.2% error tolerance (blue lines) in 3D, corresponding to the following panels in Fig. 4: (A) Fig. 4B; (B) Fig. 4C; (C) Fig. 4J; (D) Fig. 4F; (E) Fig. 4G; and (F) Fig. 4M. The corresponding parameter estimate for *N* is displayed on each plot. A, B, D and E correspond to short dashes for which almost the entire flight was modelled from target launch to intercept. Gridlines are at 10 m spacing.

To check whether PN guidance could also capture the altitudinal component of the gyrfalcon flights, we tried re-fitting all of the independently fitted PN simulations in 3D (Fig. 5). Although the number of flights that could be modelled successfully under PN dropped to 12/20 in 3D, comprising 734 m and 69.4 s of flight fitted at 1.2% error tolerance, the parameter estimates in the *n*=12 successfully fitted 3D simulations (median *N*=1.0; 1st, 3rd quartiles: 0.2, 1.4) were similar to those of the same flights in 2D (median *N*=1.2; 1st, 3rd quartiles: 0.6, 1.8), and were not significantly higher or lower in either case (sign test: *P*=0.39). It should be noted, however, that the flight path was usually quite shallow (Fig. 5), and that GPS measurement error is expected to be higher in the vertical than in the horizontal. We therefore focus the remainder of our reporting on the 2D simulations.

### PN models both short dashes and extended chases in the terminal phase

The 20 gyrfalcon flights comprised 10 short dashes lasting from 3 to 7 s (Fig. 4A–I), and 10 extended chases lasting from 18 to 223 s (Fig. 4J–R). Typically, these short dashes correspond to the birds' maiden flights (Table S1), whereas the extended chases correspond to their second flights (Table S1), which is because the pilot made less of an attempt to evade capture on the maiden flight. We found no evidence of any systematic increase or decrease in the independently fitted parameter estimates for *N* between the gyrfalcons' maiden and second flights, for the small subsample of *n*=6 individuals for which paired data were available (sign test: *P*=0.69). Of the short dashes, 7/10 were modelled successfully from target launch to intercept (Fig. 4A–G), whilst 2/10 were modelled successfully for two-thirds of the total distance flown (Fig. 4H,I). For the 9/10 extended chases that were modelled successfully (Fig. 4J–R), the simulations only captured the terminal phase of the attacks (median duration fitted: 8.2 s; 1st, 3rd quartiles: 3.1, 12.3 s). This nevertheless represents a substantial amount of flight fitted by distance (median distance fitted: 86.9 m; 1st, 3rd quartiles: 19.9, 146.5 m), because of the high speeds reached by the end of a chase.

### Naive gyrfalcons operate at lower navigation constants than experienced peregrine falcons

All 13 flights from our published dataset on experienced peregrine falcons (Brighton et al., 2017) could be modelled successfully in 2D under PN (Fig. S3, Table S3), compared with only 11/13 under PP (Table S3), when estimating the guidance parameters independently for each flight. Whereas the PN simulations for the peregrine falcons fitted 1517 m and 99.6 s of flight at 1.0% error tolerance in 2D, the PP simulations fitted only 927 m and 57.8 s (Table S3), which confirms our previous finding that PN is the better supported of the two pure guidance laws for these data (Brighton et al., 2017). The independently fitted PN+PP simulations for the peregrine falcons fitted 1740 m and 131.2 s of flight successfully at 1.0% error tolerance in 2D, but as the parameter estimates for *K* were inconsistently signed (two-tailed sign test: *P*=0.10; *n*=13 flights; Table S3), in contrast to the consistently positive parameter estimates for *N* (two-tailed sign test: *P*<0.001), any apparent improvement over PN is likely to be attributable to overfitting. This conclusion is confirmed by the globally fitted simulations maximizing the total distance of flight fitted at 1.0% error tolerance over all flights, because the PN+PP simulations at the global optimum of *N*=2.4 and *K*=0.2 fitted scarcely any more flight data (994 m and 65.2 s of flight from 11/13 flights) than the PN simulations at the global optimum of *N*=3.0 (976 m and 63.2 s of flight from 10/13 flights). In contrast, the PP simulations at the global optimum of *K*=1.0 fitted only 625 m and 36.6 s of flight from 4/13 flights. It follows that PN is the best supported of the three guidance laws in peregrine falcons. This is the same conclusion as we reached above for gyrfalcons, and it therefore makes sense to compare the tuning of the PN guidance law between these species.

**Fig. 6.**
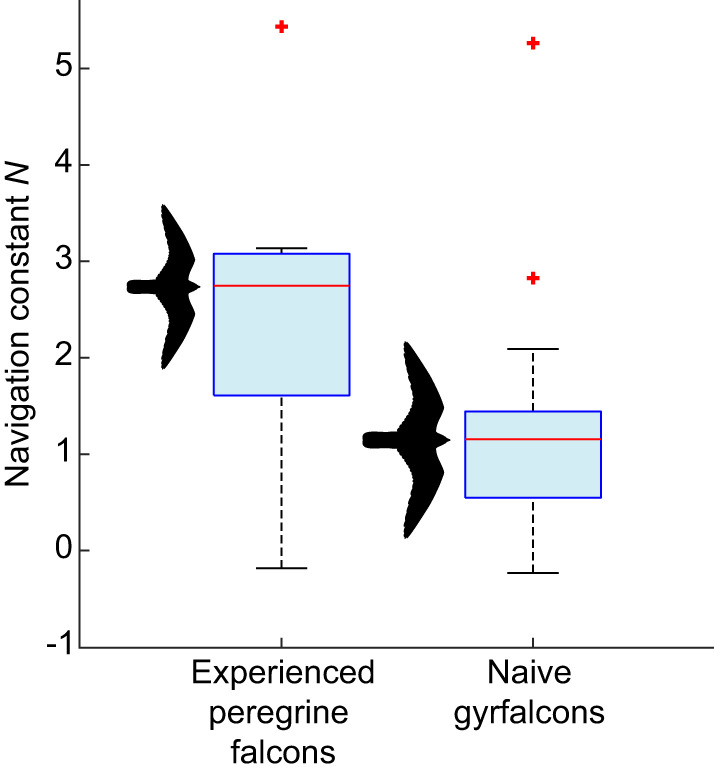
**Box-and-whisker plots comparing parameter estimates for *N* in PN guidance models fitted independently to the 13 attack flights from *n*=4 peregrine falcons and 18 attack flights from *n*=13 naive gyrfalcons, all in pursuit of manoeuvring targets.** The centre line of each box denotes the median for all flights; the lower and upper bounds of the box denote the 1st and 3rd quartiles; crosses indicate outliers falling >1.5 times the interquartile range beyond the 1st or 3rd quartile (one extreme outlier for the peregrine falcons not shown); whiskers extend to the farthest datapoints not treated as outliers.

**Fig. 7.**
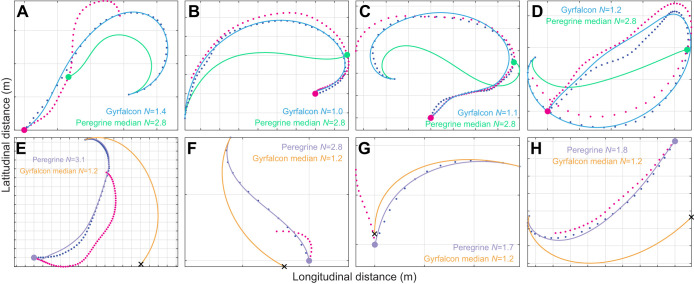
**Effect of the navigation constant *N* on the dynamics of PN guidance.** (A–D) Selection of 4 successfully modelled gyrfalcon flights involving substantial turning, showing the measured trajectory of the target (magenta dots) and attacker (blue dots) overlain with the best-fitting trajectory under PN guidance at the value of *N* displayed on the panel (blue line), and with the trajectory that would have been followed for the same initial conditions and target motion at the median value of *N*=2.8 for peregrine falcons (green line). Green circle shows the predicted point of intercept had the gyrfalcon used the median value of *N* for peregrine falcons; note that this is always sooner than the actual point of intercept (magenta circle). (E–H) Selection of 4 successfully modelled peregrine falcon flights involving substantial turning, showing the measured trajectory of the target (magenta dots) and attacker (blue dots) overlain with the best-fitting trajectory under PN guidance at the value of *N* displayed on the panel (lilac line), and with the trajectory that would have been followed for the same initial conditions and target motion at the median value of *N*=1.2 for gyrfalcons (orange line). Lilac circle shows the actual point of intercept; black cross shows the predicted position of the bird had the peregrine falcon used the median value of *N* for gyrfalcons; note that this is always at some significant distance from the target. Panel letters correspond to the following panels in Fig. 4 and Fig. S3: (A) Fig. 4M; (B) Fig. 4P; (C) Fig. 4Q; (D) Fig. 4R; (E) Fig. S3-5B; (F) Fig. S3-S3D; (G) Fig. S3-S3J; and (H) Fig. S3-5C. Gridlines are at 10 m spacing.

The independently fitted parameter estimates for *N* in the gyrfalcons (median *N*=1.2; 1st, 3rd quartiles: 0.5, 1.4) were systematically lower (Fig. 6) than those for the peregrine falcons (median *N*=2.8; 1st, 3rd quartiles: 1.6, 3.1). This difference was statistically significant (Mood's median test), when treating repeated measures from the same individual as independent datapoints (χ^2^_1__,*n*=31_=7.30; *P*=0.007), and when analysing only the median values of *N* for each individual to eliminate pseudo-replication (χ^2^_1,*n*=16_=5.33; *P*=0.02). The same pattern was observed in the globally fitted simulations, with a global optimum of *N*=1.1 in the gyrfalcons, compared with a global optimum of *N*=3.0 in the peregrine falcons. The statistically significant difference in the distribution of *N* between the two species has a profound effect on the chase dynamics, as can be shown by simulating the intercept trajectories that the gyrfalcons would have followed had they used the median value of *N* for the peregrine falcons, and vice versa (Fig. 7). It is clear by inspection that the gyrfalcons would have intercepted the target sooner had they followed the trajectory commanded at the median value of *N*=2.8 for peregrine falcons, and conversely that the peregrine falcons would not have intercepted the target as soon as they did had they followed the trajectory commanded at the median value of *N*=1.2 for the gyrfalcons. This begs the question of why the gyrfalcons operated at such low values of *N* at all, which we tackle from various perspectives in the Discussion, having first considered our key findings and their limitations.

## DISCUSSION

### Key scientific findings and their limitations

There are two key findings of this work: first, that naive gyrfalcons chase aerial targets as if using the same PN guidance law as was found previously in experienced peregrine falcons (Brighton et al., 2017); and second, that they do so at a lower value of the navigation constant *N* (Fig. 3). The first of these key findings is demonstrated by showing that PN models the data more successfully than PP, and more economically than PN+PP, in simulations using guidance parameters fitted independently to each flight and globally across all flights. It is of course plausible that the data might be even better modelled by some alternative guidance law that we have not yet tested, but as there are only a limited number of variables that can be fed back to command steering in a particle model of interception, we think it likely that any such guidance law would be a variant of PN. The second of our key findings is demonstrated by using non-parametric statistics to confirm a systematic difference in the independently fitted parameter estimates for the guidance constant *N* between the gyrfalcons (median *N*=1.2; 1st, 3rd quartiles: 0.5, 1.4) and peregrine falcons (median *N*=2.8; 1st, 3rd quartiles: 1.6, 3.1), for identically analysed data collected using identical GPS loggers. The same difference is seen in the globally fitted parameter estimates for the gyrfalcons (*N*=1.1) and the peregrine falcons (*N*=3.0). The primary limitations of these conclusions are that the experiments with peregrine falcons used a towed lure rather than the Roprey model used with the gyrfalcons, and that the sample of peregrine falcons comprised only *n*=4 individuals. However, as we have previously reported similar navigation constants (median *N*=2.6; 1st, 3rd quartiles: 1.7, 3.3) in an independent sample of *n*=3 experienced peregrine falcons attacking stationary targets (Brighton et al., 2017), we are confident that our results can be generalized.

### Technical limitations

It is important to emphasize that we have not necessarily identified a unique navigation constant *N* for either species, albeit this is how the data are modelled in the globally fitted simulations. Rather, what the independently fitted simulations definitively show is that there are distinct intervals within which the respective values of *N* for each species typically fall. Nevertheless, it is important to note that whilst we have reported a unique estimate of *N* for each flight, a different parameter estimate would have been reported had a different start point been chosen for the simulation. The selected start points are those which maximize the amount of flight fitted at the specified error tolerance, and therefore represent an objective compromise between goodness of fit and amount of data modelled. It is therefore uncertain how much of the resulting variability in our parameter estimates for *N* is the result of measurement error as opposed to genuine behavioural flexibility. In particular, the use of GPS loggers recording speed and position at 5 Hz precludes perfect synchronization of the attacker and target trajectories and hence estimation of the sensorimotor delay, so it is plausible that data collected at higher spatiotemporal precision would show lower variability in the parameter estimates for *N*. Finally, our modelling only accounts for the influence of wind insofar as the simulated groundspeed is matched to that of the measured groundspeed. The simulations therefore treat any distortions of the bird's track due to the wind as if these were produced by the attacker's own steering commands, although the effect of this is mitigated in close pursuit by the fact that the attacker and its target are subject to the same wind. Nevertheless, gusting winds might explain some of the more prominent inflections in the measured flight trajectories that are not captured by the simulations fitted at higher wind speeds (Fig. 4R), or at greater horizontal (Fig. 4J) or especially vertical separation (Fig. 5C) between the attacker and its target. Altitudinal variation in wind speed may therefore explain the poorer fit of some of the 3D simulations (Fig. 5).

### Physical and physiological constraints

For a given line-of-sight rate, the higher values of *N* in peregrine falcons are expected to command turning at approximately twice the angular rate of the lower values of *N* in gyrfalcons. Minimum turn radius scales linearly with wing loading, *W* (Taylor and Thomas, 2014), which we estimate to be ∼6.0 kg m^−2^ in gyrfalcons compared with ∼5.3 kg m^−2^ in peregrine falcons, based on the species means of measurements from other captive individuals (Pennycuick et al., 1994). Gyrfalcons are therefore expected to be less manoeuvrable than peregrine falcons, with a ∼13% larger minimum turn radius. They are also expected to be less agile, because flight speed scales as *W*^1/2^ (Taylor and Thomas, 2014), such that maximum turn rate scales as *W*^−1/2^, and is therefore expected to be ∼6% lower in gyrfalcons than in peregrine falcons. In principle, this physical constraint could force the navigation constant *N* to be lower in gyrfalcons, but the expected difference in their agility is too small to explain the twofold difference in *N* that we observe. Furthermore, there is no evidence that physical constraints on turning influenced the shape of the recorded trajectories. Rather than turning as tightly as possible on a circular arc, the birds instead followed a curved trajectory of increasing or decreasing radius, characteristic of the time history of turning commanded under PN guidance at higher or lower values of *N*, respectively (Fig. 4). The observed flight trajectories are similarly inconsistent with an old hypothesis proposed by Tucker (2000), who argued that the curved attack trajectories of falcons could be generated by steering so as to hold the target's image on the laterally directed central fovea of the left or right eye whilst holding the head in line with the body (Tucker et al., 2000). This constraint is expected to produce a trajectory in which the deviation angle δ between the attacker's velocity and its line-of-sight to target is held constant at approximately ±40 deg, but there is no evidence of this from the data (see Fig. 2; see also Kane and Zamani, 2014).

### A functional account of the observed variation in navigation constant

In principle, the variation in *N* within and between species (Fig. 6) might be explained as a behavioural response under linear-quadratic optimal guidance theory (Shneydor, 1998; Siouris, 2004), which predicts an optimum value of *N*=3*v*_c_/(*v*cosδ) for attacks on non-manoeuvring targets minimizing overall steering effort. In practice, the median values of the ratio *v*_c_/(*v*cosδ) for each flight did not differ significantly between the two species (χ^2^_1, *n*=31_=1.55; *P*=0.21), and nor were they significantly related to *N* in a bisquare robust regression controlling for species (*t*_28_=0.46; *P*=0.69). We therefore found no evidence that the statistically significant difference in the parameter estimates for *N* between the peregrine falcons and gyrfalcons reflects a direct functional response to variation in the rate at which they closed range on the target relative to their own approach speed. This conclusion is based on classical theory for non-manoeuvring targets, and therefore takes no account of the target's manoeuvres. Nevertheless, the tortuosity (*T*) of the lure's path, defined as its overall path length divided by the straight line distance from start to finish, was similar in the experiments with peregrine falcons (median *T*=1.6; 1st, 3rd quartiles: 1.3, 2.4) and gyrfalcons (median *T*=1.7; 1st, 3rd quartiles: 1.3, 2.5), so we conclude that differences in target manoeuvres are unlikely to explain the variation in *N* that we observed between species. Even so, an identical target manoeuvre will produce a greater angular change in the line-of-sight vector the closer the attacker is to its target, thereby demanding a higher rate of turning under PN. This is important, because whereas the gyrfalcons took off together with the target at a range of ∼20 m, the peregrine falcons were launched separately and sometimes at a range of >100 m. When operating at close range, PN guidance is less prone to being thrown off by erratic target manoeuvres if *N* is low (Brighton and Taylor, 2019), so it is plausible that the lower values of *N* found in the gyrfalcons might reflect a behavioural response to their proximity to the target at the initiation of the attack.

### An adaptive account of the observed variation in navigation constant

A complementary adaptive argument can also be made at the species level. Whereas peregrine falcons have a very flexible diet (Cade, 1982), gyrfalcons depend heavily on a single prey type, with ptarmigan (*Lagopus* spp.) comprising 74% of the catch on average across 17 studies recording 66,726 individual prey items over most of the gyrfalcon's range (Nielsen and Cade, 2017). Ptarmigan have a high wing loading of ∼9.7 kg m^−2^ (Greenewalt, 1962), which is ∼62% higher than that of a gyrfalcon. They are therefore intrinsically fast fliers, with large flight muscles that provide rapid acceleration in an explosive take-off, and short wings that make them less well adapted to sustained aerobic flight (Pennycuick et al., 1994). The prolonged tail-chasing behaviour that is typical of gyrfalcons (Cade, 1982) is therefore thought to serve to tire their prey and allow capture (Pennycuick et al., 1994). Such behaviour is promoted by the low values of *N* found in gyrfalcons (Fig. 7), because PN with a low navigation constant *N*≈1 commands turning at an angular rate 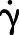 approximately equal to the line-of-sight rate 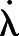. Once a tail chase has been initiated, this will tend to keep the attacker flying behind its target, and it will not be thrown off too far by the erratic jinking manoeuvres that are characteristic of the evasive flight of ptarmigan and many other prey (Mills et al., 2018, 2019). The low values of *N* found in gyrfalcons therefore make sense from an adaptationist perspective if the function of their PN guidance is to pursue the prey doggedly until it tires. This is in contrast to the higher values of *N* found in peregrine falcons, which make sense if the function of their PN guidance is to exploit the speed and manoeuvrability acquired through stooping in order to intercept prey quickly and efficiently (Mills et al., 2018, 2019).

### Evolutionary and ontogenetic implications

We found no evidence that the value of *N* changed systematically between the gyrfalcons' first and second flights, but it is reasonable to expect that the same birds would have learned to tune their guidance over longer time scales. For example, although wild gyrfalcons do not usually stoop, falconers commonly train captive birds to do so (Tucker et al., 1998), which implies a degree of behavioural flexibility in their guidance. Likewise, whereas naive gyrfalcons tend to fly directly at their prey, experienced birds seem to anticipate their prey's behaviour. This being so, we cannot exclude the possibility that naive peregrine falcons – and perhaps other species of bird using aerial pursuit – might use low-gain PN initially and increase their navigation constant *N* through experience. Even so, our finding that the maiden attack flights of naive gyrfalcons are well modelled under PN guidance strongly suggests that this behavioural algorithm is embedded in an innate guidance pathway involving the oculomotor system, neck motor system and vestibular system. Moreover, the fact that the same form of guidance law also models the attack flights of experienced peregrine falcons is consistent with the hypothesis that this innate guidance pathway is ancestral to the clade comprising the peregrine falcons and hierofalcons, of which the gyrfalcon is a member (Wink, 2018). Formal confirmation of this hypothesis would require the same behavioural algorithm to be identified in inexperienced individuals of another hierofalcon, or in a close outgroup such as the merlin or hobby falcons, both of which specialize in aerial pursuit. However, as Harris' hawks have also been found to use a PN element in their mixed PN+PP guidance law (Brighton and Taylor, 2019), we hypothesize that an innate guidance pathway using line-of-sight rate to steer motion towards a target may have originated much deeper in the avian phylogeny and perhaps even in non-avian theropods.

Pursuit behaviours that might also be generated by low-gain PN have been observed in humans catching balls (Oudejans et al., 1999) and in dogs catching frisbees (Shaffer et al., 2004), although whether mammals use PN guidance to implement their pursuit – and if so, whether they have evolved to do so independently – remains to be explored. In any case, because PN guidance commands turning in proportion to the line-of-sight rate of the target, which is necessarily defined in an inertial frame of reference, we would expect other animals using PN to share a common sensorimotor architecture that fuses sensory input from the visual and vestibular systems to obtain the line-of-sight rate, and that uses the resulting signal to generate motor commands. Alternatively, it is possible that the distant landscape might serve as a visual proxy for the inertial frame of reference in aerial hunters, with target motion being measured directly with respect to the visual background (see also Kane and Zamani, 2014). It would therefore be of particular interest to test whether PN guidance also models the attack behaviours of the more distantly related kestrels, given their specialism on terrestrial prey with no distant visual background against which to assess prey motion.
